# What supports sustainable pace in the operating room? A sequential explanatory mixed-methods study using self-determination theory

**DOI:** 10.1093/intqhc/mzag130

**Published:** 2026-09-09

**Authors:** Johan Eriksson, Kristina Lämås, Lena Lindholm, Micael Appelblad, Malin Sund

**Affiliations:** Department of Diagnostics and Intervention/Surgery, Umeå University, Umeå, Sweden; Department of Nursing, Umeå University, Umeå, Sweden; Heart Center, University Hospital of Umeå, Umeå, Sweden; Department of Public Health and Clinical Medicine, Umeå University, Umeå, Sweden; Department of Diagnostics and Intervention/Surgery, Umeå University, Umeå, Sweden; Department of Surgery, University of Helsinki and Helsinki University Hospital, Helsinki, Finland

## Abstract

**Background:**

Operating-room productivity is commonly pursued through throughput metrics, yet frontline staff point to organizational and interpersonal conditions that make a working pace sustainable. How everyday practices relate to staff motivation, and thereby to dependable workflow, remains underexplored.

**Research question:**

(1) What organizational and interpersonal conditions support or thwart a sustainable work pace among perioperative nursing staff? (2) How can these conditions be understood through Self-Determination Theory (SDT)?

**Methods:**

We used a sequential explanatory mixed-methods design at two operating room units in a Swedish university hospital. In Phase 1 the Safety Attitudes Questionnaire–Operating Room version was administered (October 2022; *n* = 47) to profile contextual contrasts between units. In Phase 2, semi-structured interviews (December 2022–March 2025; *n* = 24) were analyzed with inductive qualitative content analysis, after which SDT was used to interpret the resulting categories. Qualitative reporting followed the COREQ.

**Results:**

Survey responses differed between units in safety climate (32% vs. 68%; *P *= .02), stress recognition (57% vs. 21%; *P *= .02) and perceptions of management (18% vs. 63%; *P *= .002). Interviews yielded four cross-cutting conditions: predictable workflow and usable tools; psychologically safe teamwork; participatory planning with visible follow-through; and learning structures for mastery, with workload as a boundary condition. The same families of practice appeared at both units but were described with differing emphasis. Interpreted through SDT, these conditions supported autonomy, competence and relatedness when present and thwarted them when absent; need-thwarting patterns (late list changes, parallel documentation, sharp communication, tokenistic consultation) co-occurred with workarounds and conditional intentions to stay.

**Conclusion:**

Among perioperative nursing staff, a sustainable pace was described less as working slowly than as predictable routines, professional judgement over timing, stable teamwork, and protected standards of care under pressure. Modest, auditable routines that jointly support autonomy, competence and relatedness offer transferable, hypothesis-generating design principles for dependable operating-room productivity, rather than a ranking of units.

## Introduction

Efforts to raise operating room (OR) productivity often concentrate on throughput indicators such as utilization, on-time starts and turnover time [1]. OR staff have also described efficiency in broader terms that incorporate teamwork, quality of care and patient benefit [2]. These indicators describe outputs rather than the conditions that make a dependable pace possible in everyday work [3]. When planning, staffing, tools and team interaction are misaligned, teams compensate with workarounds that restore flow transiently while eroding vigilance, learning and morale [4, 5].

Self-Determination Theory (SDT) holds that three basic psychological needs (autonomy, competence and relatedness) drive motivation and adaptive functioning when supported, and defensive, compliance-based effort when frustrated [6, 7]. In perioperative care, SDT complements work on non-technical skills, psychological safety and standardized communication by specifying how practices such as participatory planning, protected breaks and brief/debrief routines shape motivation and coordination [5, 8, 9].

Much OR productivity research prioritizes scheduling algorithms and aggregate utilization, leaving frontline perspectives on how everyday practices relate to need support or thwarting underexplored [1]. Comparative designs risk producing unit ‘league tables’, whereas practitioners need transferable design principles independent of site. A mechanism-oriented synthesis linking familiar problems (late list changes, equipment bottlenecks, rota volatility) to the three needs and to observable consequences (restarts, rework, silence) can help close this gap [3, 8].

This study therefore addressed two research questions: what organizational and interpersonal conditions support or thwart a sustainable work pace among perioperative nursing staff, and how can these conditions be understood through SDT? We used a sequential explanatory mixed-methods design at two OR units selected for contrasting staffing models and workflows; an SAQ-OR survey provided contextual signals that informed purposive qualitative sampling, followed by interviews interpreted through SDT.

## Materials and methods

### Design and overall approach

A sequential explanatory mixed-methods design was used [10]. An SAQ-OR survey (October 2022) provided contextual signals that guided purposive interview sampling and probe development. Semi-structured interviews were conducted in two phases (December 2022–March 2025). This extended timeframe introduced the possibility of organizational change; findings were therefore interpreted with plausibility-oriented language rather than as claims of uniformity. SDT informed the interview focus and the cross-unit synthesis. Qualitative reporting followed the Consolidated Criteria for Reporting Qualitative Research (COREQ).

### Patient and public involvement

This study did not include formal patient or public involvement. It focused on staff experiences and organizational mechanisms; future work should incorporate patient perspectives on how these mechanisms affect care quality.

### Setting

Two OR units within a Swedish university hospital were studied, labelled Unit A and Unit B (labels randomly assigned) to preserve anonymity. Both provided 24/7 coverage with mixed elective and emergency caseloads. Unit A employed approximately 80 total staff across professional groups, including nursing staff, anaesthesiologists and other OR personnel; Unit B had roughly half that complement across four ORs. Staffing patterns and scheduling flexibility differed notably.

### Participants and eligibility

Eligible perioperative nursing staff, nurse anaesthetists, OR nurses and assistant nurses, were invited to the SAQ-OR survey. Surgeons, anaesthesiologists, perfusionists and surgical technologists were not included. The study was designed to examine the perspectives of the nursing roles that carry continuous responsibility for intraoperative workflow and team coordination, and this scope is reflected in the framing of the findings (see Strengths and limitations). For interviews, maximum-variation sampling was used across nurse anaesthetists and OR nurses at both units. Inclusion criteria were at least six months at the unit and at least one year in the role. All invited staff consented; no incentives were offered. Recruitment was through staff briefings and postings and direct approach on site. Interviewing began at Unit B and was later extended to Unit A to incorporate a contrasting perioperative nursing context; recruitment was concluded when further interviews added little to the established categories, on the basis of information power rather than a predetermined sample size [11]. Interview eligibility was limited to registered operating-room nurses and nurse anaesthetists, a subset of the staff surveyed; assistant nurses, eligible for the survey, were not interviewed.

### Data collection

The SAQ-OR (validated Swedish version) covers six domains: safety climate, teamwork, job satisfaction, working conditions, stress recognition and perceptions of management [12]. Interviews were conducted in Swedish by the first author (a registered nurse and doctoral researcher) in private rooms on site, and were audio-recorded and transcribed verbatim. The first two interviews included a non-managerial observer for procedural credibility. Brief field notes on participant mood and interview atmosphere were made during and immediately after each interview. Interviews lasted 38 to 75 minutes (median 61). The guide probed meanings of productivity, planning and flow, staffing and breaks, collaboration, learning, and enablers and barriers, and was refined iteratively to follow emerging patterns.

### Reflexivity

The first author is a male registered nurse with perioperative experience who works part-time as a clinician at the study hospital. Most participants were therefore known to the interviewer through shared workplace context; all were informed of his dual clinician–researcher role and that the study explored experiences rather than evaluating individuals or units. This insider position facilitated rapport but may have introduced familiarity bias. To mitigate this, probes were deliberately mechanism-focused rather than evaluative, and co-authors from varied professional backgrounds adjudicated category proposals throughout.

### Analysis

Survey domain scores were dichotomised (proportion positive, at least 4 of 5) and compared between units with two-sided Fisher’s exact tests (*α* = 0.05, unadjusted for multiple comparisons), performed in R version 4.5.1 (R Core Team, Vienna, Austria). Qualitative data management and coding were conducted in Microsoft Excel; no dedicated qualitative-analysis software was used. Interviews were analyzed using qualitative content analysis [13, 14]. Coding was inductive through a three-level manifest hierarchy: subcategories, categories and main categories, with abstraction levels kept consistent [15]. To avoid ambiguity, we use ‘subcategory’ for the most concrete practice-level grouping, ‘category’ for the middle level, and ‘main category’ for the four cross-cutting conditions; Table 2 illustrates this movement for representative meaning units. One researcher performed initial coding; one transcript was independently double coded to calibrate the approach; interim codebooks were circulated to all co-authors for adjudication.

The three components were integrated through explanatory sequencing rather than statistical combination: Phase 1 contrasts were used to focus Phase 2 sampling and probing, and Phase 2 accounts were used to explain the Phase 1 contrasts. Points where survey signals and interview accounts converged were treated as supportive; divergences were treated as informative and examined directly.

After inductive coding, SDT was used to interpret the categories. Coded segments were annotated for the need engaged (autonomy, competence or relatedness) and whether the practice appeared need-supportive or need-thwarting. We describe this step as abductive rather than deductive because we did not specify and test SDT-derived hypotheses, nor did we treat the categories as arising from data alone; instead, we moved iteratively between the inductively derived patterns and SDT to identify the account that best explained them, revising the mapping where the data resisted it. For example, the divergent SAQ-OR profiles would, deductively, predict correspondingly divergent interview accounts between units; the interviews instead showed the same families of practice at both units, described with differing emphasis. This convergence prompted us to reinterpret the units as replicate observations of shared mechanisms rather than as entities to be ranked, an inference to the best explanation that neither pure induction nor hypothesis-testing would have produced. We use ‘narrative density’ to denote this differing emphasis: the relative frequency and elaboration with which a given practice family was described at a unit, as distinct from its binary presence or absence. Analytical memos were written per unit and informed the cross-unit synthesis.

### Ethics

Approved by the Regional Ethical Review Board in Umeå, Sweden (Ref. 2017/463-31), the predecessor of the Swedish Ethical Review Authority. Written informed consent was obtained from all participants. Trustworthiness was strengthened through multi-analyst category refinement, attention to discrepant cases, an audit trail and reflexive discussion [15, 16]. Data were GDPR-compliant; transcripts were pseudonymized, with re-identification keys stored offline on encrypted media. Formal member checking was not performed and transcripts were not returned to participants for comment or correction. Consistency between raw data and reported findings was maintained by systematically cross-referencing anchor quotes against category definitions.

Because the study involved small professional groups within two identifiable OR units, unit attribution for individual quotes was omitted to reduce the risk of deductive disclosure. Unit-level differences are reported only in aggregate.

## Results

### Participants

A total of 24 perioperative nurses were interviewed, eight at Unit A and 16 at Unit B. All were nurse anaesthetists or OR nurses; reported OR experience ranged from 3 to 26 years (median 9).

### Phase 1: SAQ-OR survey

Across both units, 47 staff responded to the SAQ-OR survey (Unit A *n* = 28; Unit B *n* = 19). Based on staffing records from the survey period, 71 eligible perioperative nursing staff were employed at Unit A and 32 at Unit B, including OR nurses, nurse anaesthetists and assistant nurses. This corresponded to approximate response rates of 39% and 59%, respectively, and 46% overall. Because the denominator was based on staffing records rather than confirmed individual receipt of the questionnaire, and because distribution depended on local access and work schedules, these figures should be interpreted as approximate response rates among eligible staff. We had no systematic information on non-respondents. For these reasons, Phase 1 is treated as a source of contextual signals rather than as a representative estimate.

Between-unit differences reached significance for safety climate (32% vs. 68%; *P *= .02), stress recognition (57% vs. 21%; *P *= .02) and perceptions of management (18% vs. 63%; *P *= .002). Teamwork (64% vs. 53%), job satisfaction (93% vs. 84%) and working conditions (43% vs. 37%) did not differ significantly (Table 1). These contrasts were treated as contextual signals guiding interview focus rather than as stable performance ratings.

**Table 1:** Safety attitudes questionnaire-operating room version results by unit.^a^

Domain	Unit A (*n* = 28), *n* (%)	Unit B (*n* = 19), *n* (%)	*P*-value (Fisher’s exact)
Safety climate	9 (32)	13 (68)	.02
Stress recognition	16 (57)	4 (21)	.02
Perceptions of management	5 (18)	12 (63)	.002
Teamwork	18 (64)	10 (53)	.55
Job satisfaction	26 (93)	16 (84)	.38
Working conditions	12 (43)	7 (37)	.77

a Number and proportion of respondents scoring positive (at least 4 of 5) by SAQ-OR domain, presented as *n* (%). Denominators are the number of respondents at each unit. *P*-values are from two-sided Fisher’s exact tests, unadjusted for multiple comparisons. Contrasts were treated as contextual signals rather than performance ratings. Survey October 2022; interviews December 2022–March 2025.

**Table 2:** From meaning unit to category, with SDT interpretation.^a^

Meaning unit	Condensed meaning unit	Code	Subcategory	Main category	SDT
‘We are always planning for perfect conditions that never exist, without any buffers. When reality strikes, we have to improvise and compensate.’ (P18)	Planning assumes perfect conditions; no buffers; improvization when reality differs	Unrealistic planning assumptions	Poor planning/system collapse	Predictable workflow and usable tools	C−, A−
‘Do you have this open climate, that you dare to tell a surgeon who you think is doing something crazy… I probably do.’ (P4)	Open climate enables speaking up across hierarchy	Voice across professional hierarchy	Hierarchy lowers safety	Psychologically safe teamwork	R+, C+
‘I have education, experience, I see things from my perspective that others might miss. But that knowledge is not used.’ (P23)	Expertize present but not utilized	Underused frontline expertize	Lack of influence and participation	Participatory planning and visible follow-through	A−, R−
‘New colleagues are thrown into the thick of it, without a proper introduction.’ (P20)	Insufficient onboarding for new staff	Inadequate onboarding	Supervision/onboarding gaps	Learning structures to mastery; load as boundary	C−

a Meaning units are verbatim extracts; condensed meaning units summarize the essential meaning; codes, subcategories and main categories reflect successive abstraction levels [13, 15]. The final column shows the abductive SDT interpretation: the basic need(s) engaged (autonomy, A; competence, C; relatedness, R) and whether the practice appeared need-supportive (+) or need-thwarting (−).

### Phase 2: interviews

**Figure 1:**
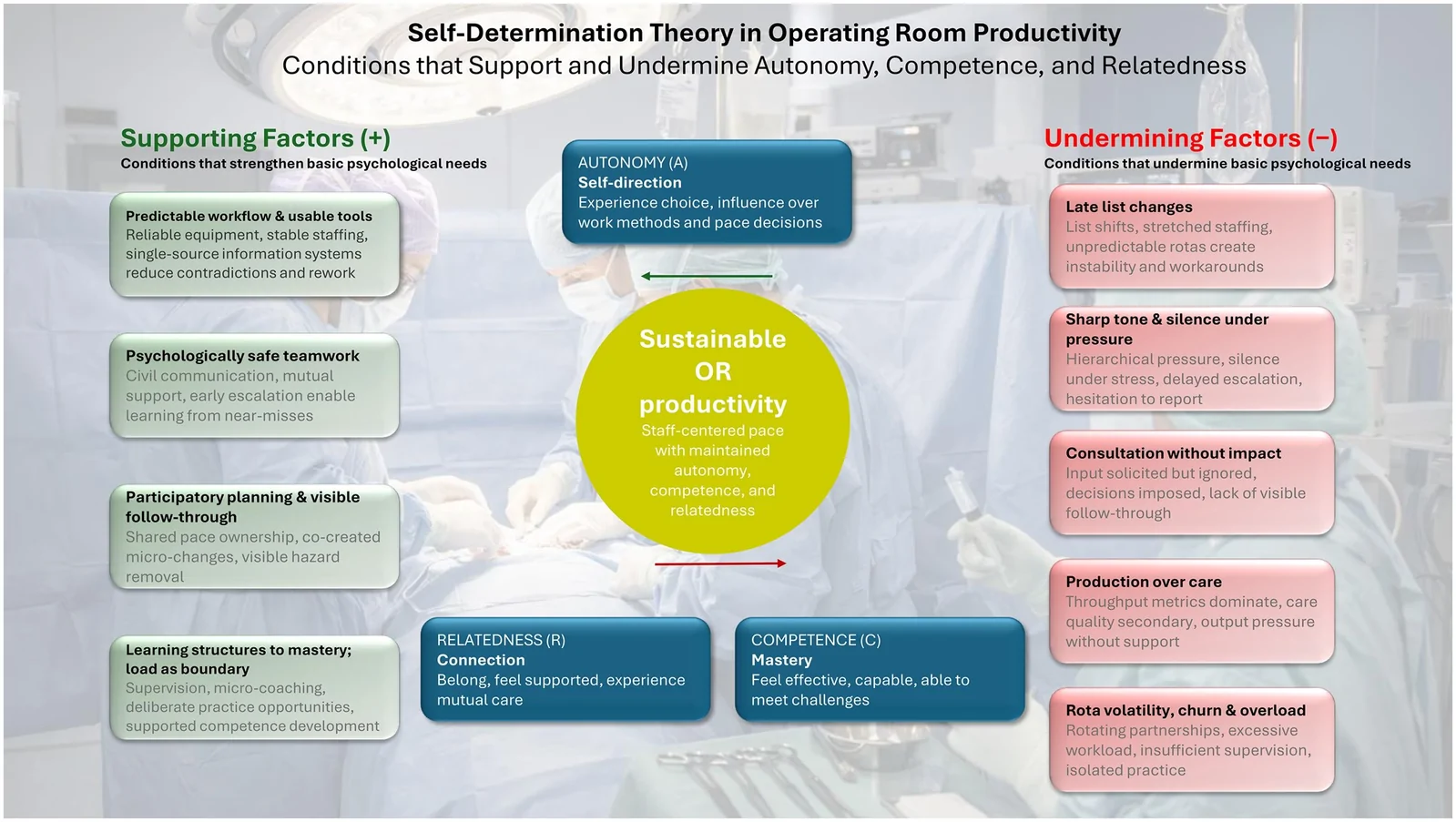
Conceptual summary of need-supportive and need-thwarting conditions for a sustainable OR pace, organized by the three basic psychological needs in SDT (autonomy, competence, and relatedness). Supporting factors are shown on the left; undermining factors on the right.

Four cross-cutting conditions described how a sustainable OR pace was experienced and maintained across both units. The same families of practice appeared at both sites, with differing narrative density rather than binary presence or absence. Table 2 illustrates how representative meaning units were condensed, coded, grouped and interpreted through SDT; Fig. 1 provides a conceptual overview; Table 3 summarizes selected subcategories.

**Table 3:** Cross-cutting main categories and selected central subcategories.^a^

Predictable workflow and usable tools	Psychologically safe teamwork	Participatory planning and visible follow-through	Learning structures to mastery; load as boundary
Poor planning/system collapse	Destructive communication and insecurity	Lack of influence and participation	Physical and mental exhaustion
Parallel systems to compensate	Silence—imposed/normalized	Engaged leadership and change capacity	Supervision/onboarding gaps
Technical resources and equipment	Hierarchy lowers safety	Quality improvement and patient safety	Missing space for reflection
Understaffing and pace	Respectful team culture and parity	Strategic perspective	Competence and professional growth
Information handling	Collegial support & tacit coordination	Historical development	Stress impact on care quality

a The middle (category) level is omitted; selected subcategories and main categories are shown. Full SDT overlay in Appendix 1.

To protect participant anonymity in small professional groups, illustrative quotes are attributed by participant number and professional role only, not by unit. Unit-level patterns are instead described in aggregate in the surrounding text.

### Predictable workflow and usable tools

When tools, information and staffing were reliably available, staff described a steadier tempo, fewer resets and earlier escalation (competence and autonomy supported); scarcity or unpredictability generated workarounds and reactive problem-solving (competence and autonomy thwarted). Unit A narratives centred on resource constraints and organizational fragility (material limits, understaffing, parallel workaround systems, production pressure), whereas Unit B more often described adequate resources and standardized working, though similar fragility emerged when these lapsed.

*‘We are always planning for perfect conditions that never exist, without any buffers. When reality strikes, we have to improvise and compensate.’* (P18, Nurse anaesthetist)

*‘Structured, open and forward-thinking. Colleagues anticipate needs and act before you even have to ask for it.’* (P11, OR nurse)

### Psychologically safe teamwork

Civil tone, quick mutual help and approachable colleagues made it safe to speak up and kept corrections timely (relatedness and competence supported). Hierarchy gradients and sharp exchanges were associated with silence and delayed correction (relatedness and competence thwarted). In Unit B, social cohesion and a shared focus on the patient were frequently emphasized; when a production-only framing dominated, staff described less room for voice and reflection.

*‘When there are tensions and hierarchies, communication becomes poorer, and this can affect both efficiency and safety.’* (P23, OR nurse)

*‘For me, there is a reason why the OR table is in the middle of the room. That is what we should rally around, and everyone should work together.’* (P10, Nurse anaesthetist)

*‘When it feels like it’s just production… then you lose why you do it.’* (P20, Nurse anaesthetist)

### Participatory planning and visible follow-through

Ownership rose when staff had a voice in plans, rationales were shared and known blockers were closed visibly (autonomy and relatedness supported). Tokenistic consultation and unexplained changes eroded trust and initiative (autonomy and relatedness thwarted). A minimal but meaningful participatory signal was described as being seen, listened to and met with a genuine attempt to act, mattering most when paired with visible follow-through.

*‘I have education, experience, I see things from my perspective that others might miss. But that knowledge is not used.’* (P23, OR nurse)

*‘Must understand that they [management] can’t change everything, but they listen to you. Most of the time, the management listens and tries.’* (P4, Nurse anaesthetist)

*‘It feels like management is more focused on production than on personnel issues.’* (P17, OR nurse)

In Unit B, participatory mechanisms, frontline-led quality improvement and a shared strategic perspective linking aims to patient care, were described more consistently, but production pressure reappeared when they lapsed. Across both units, late list changes without rationale generated workaround culture, whereas change windows and brief rationales let lists run calmly.

### Learning structures to mastery; load as boundary

Supervision, reflection and visible progression pathways converted effort into mastery and safer initiative (competence and autonomy supported), but chronic load set the boundary conditions: without slack and recovery, competence growth stalled and autonomy felt risky (competence and autonomy thwarted). The affective and cognitive costs of sustained pressure, including moral distress, reduced patient contact, stress-related quality slips, exhaustion and cynicism, accumulated when load was high and recovery inadequate.

*‘New colleagues are thrown into the thick of it, without a proper introduction.’* (P20, Nurse anaesthetist)

*‘There is no forum where we are allowed to vent properly, where someone actually listens.’* (P17, OR nurse)

*‘You know they’re scared… And then you just have to quickly go in and roll them away. It doesn’t feel good in my heart not to have time to really see them.’* (P18, Nurse anaesthetist)

*‘I feel confident. I know my job well. That comes from having worked for a long time.’* (P4, Nurse anaesthetist)

### Sustainability signals: motivation and retention

Across all four conditions, intrinsic motivation and love of the craft persisted, but intentions to stay tracked the extent to which everyday conditions supported the three needs. Where need-supportive conditions were present, staff expressed conditional commitment to remain; where absent, thoughts of leaving were more frequent, and staff described building informal compensatory routines of their own.

*‘If the conditions were a bit better… then I could probably see myself staying.’* (P18, Nurse anaesthetist)

## Discussion

### Statement of principal findings

The main finding was that a sustainable pace was not described simply as working more slowly or reducing throughput. Rather, perioperative nursing staff described it as the ability to maintain predictable routines, exercise professional judgement over sequencing and timing, rely on stable teamwork, and preserve standards of care under production pressure. Interpreted through SDT, these conditions appeared to support or thwart autonomy, competence and relatedness in ways that shaped whether pace was experienced as dependable or unsustainable. Across the four conditions, operational difficulties such as late list changes and parallel documentation were less the root problem than downstream consequences of whether these needs were met in everyday practice [1].

### Strengths and limitations

Strengths include a theory-informed synthesis across two units, an explicit audit trail for the SDT interpretation applied to inductively derived codes, and translation into practice-level design principles. The most important limitation is scope: the study captured the perspectives of perioperative nursing staff only. Surgeons, anaesthesiologists and perfusionists were not sampled, so the findings speak to the conditions for productivity as experienced by nursing roles rather than to OR productivity in its full multidisciplinary sense, and claims are bounded accordingly. Other limitations include the single-organization setting and a prolonged, phased data-collection window (28 months), which may reflect organizational change rather than stable between-unit differences; approximate survey response rates based on staffing records rather than confirmed individual receipt of the questionnaire, together with absent data on non-respondents, which limit the weight Phase 1 can bear; and the analytic choices inherent in qualitative content analysis, where demands on transparency grow with abstraction [17]. We present mainly the main categories, summarising selected subcategories in Table 3, so the middle category level is not exhaustively reported. The SDT interpretation is an abductive step that risks theory-imposition; we therefore separated inductive coding from SDT annotation, retained disconfirming material, and used co-author adjudication. Inter-rater reliability statistics were not calculated, and the findings are explanatory and hypothesis-generating rather than confirmatory.

The unequal distribution of interview participants between units reflected the study’s explanatory rather than comparative purpose. The two units were treated as contrasting contexts through which common mechanisms could be explored, and interviews were analyzed across units as a shared perioperative nursing context. Later interviews primarily reinforced existing categories rather than generating substantially new patterns.

### Interpretation within the context of the wider literature

Research on teamwork and communication shows that interprofessional collaboration affects safety and efficiency but rarely specifies motivational pathways [4, 5, 18]. Our SDT-framed synthesis offers a candidate mechanism: civil tone, mutual help and equitable voice supported relatedness and competence, enabling timely correction and steadier tempo, consistent with Edmondson’s work on psychological safety [8] and extended here to workflow consequences. SDT distinguishes need satisfaction from two adverse states that the perioperative literature often merges. Need frustration refers to the active undermining of a need (e.g. controlling or dismissive management); need dissatisfaction is the milder state that follows when support is simply absent [19, 20]. Our accounts contained both: tokenistic consultation and sharp correction read as frustration, whereas missing learning time or unacknowledged expertize read as unmet rather than actively thwarted need, a distinction that plausibly calls for different responses. Work on basic needs in organizations indicates that need-supportive leadership fosters autonomous motivation [7, 21, 22]; our findings add a high-acuity clinical setting in which the costs of need frustration, including silence under pressure, workaround culture and motivational fatigue, are particularly consequential, and suggest that the four conditions map onto established need-supportive levers (meaningful participation, structure and relational support) rather than constituting a separate taxonomy [23].

The SAQ-OR provided contextual signals rather than ratings, helping us sample purposively and focus on safety climate, stress recognition and perceptions of management. Interview themes largely converged with these signals: units with stronger safety-climate narratives also gave more accounts of predictable workflow and equipment readiness. Given the time between the October 2022 survey and the later interviews, we read convergence as supportive triangulation rather than confirmation. We retained the unit labels for audit purposes, but this was not a comparative evaluation: both units described the same families of practice, with narrative density, not presence or absence, the main difference, plausibly reflecting sampling timing, sample size and role mix.

### Implications for policy, practice and research

The working hypothesis is that dependable OR productivity depends less on raw speed or scheduling precision than on whether everyday routines jointly support the three basic psychological needs: autonomy, competence and relatedness. Several mutually reinforcing, auditable routines follow. Participatory list planning, with earlier list locking and documented rationales for late changes, may support autonomy. Short pre-list huddles and a brief ‘one keep/one improve’ debrief may create predictable rhythm and normalize civil correction. A single source of truth replacing parallel documentation may reduce rework, while modest scheduling buffers may dampen variability. Visible learning pathways, such as micro-coaching, brief weekly reflection and trackable competencies, may support competence under load. Framed this way, the same routines double as an audit: organizations can ask where everyday practice supports the three needs and where it thwarts them. These propositions are hypothesis-generating, grounded in staff accounts from two units, and require prospective testing before any causal claim.

## Conclusions

Among perioperative nursing staff, a dependable OR pace was less a function of raw speed or scheduling precision than of everyday conditions that jointly supported autonomy, competence and relatedness. When staff had a genuine voice in planning, when tools and information were reliably available, when communication remained civil, and when learning was protected, pace was steadier and motivation more durable. The four conditions are not independent levers but mutually reinforcing, and the modest, auditable routines outlined above appear sufficient to move conditions towards need support without large-scale reorganization. Prospective testing in other OR settings is needed to establish generalizability. The transferable design principles offered here are a practical starting point for improving sustainable OR productivity from the ground up.

## Supplementary Material

mzag130_Supplementary_Data

## Data Availability

The datasets generated and analyzed during this study are not publicly available due to participant confidentiality constraints and applicable GDPR regulations. Requests for data access may be considered by the corresponding author on a case-by-case basis.
