# Supplementary material for: What supports sustainable pace in the operating room? A sequential explanatory mixed-methods study using self-determination theory
Source: Int J Qual Health Care. 2026 Sep 9;38(3):mzag130. doi: 10.1093/intqhc/mzag130 (PMC13597115; doi:10.1093/intqhc/mzag130)
Supplement: Supplementary file 1 [file mzag130_supplementary_data.zip › Appendix_1.docx]

**Appendix 1. Main categories with SDT overlay**

Main category – Predictable workflow & usable tools

Resources & work organisation

Cost cuts that backfire – (risk: C−)

**• Support:** Smart standardisation and fit-for-purpose consumables.

**• Thwarting:** Low-quality consumables and resource decisions that create downstream friction.

**• Consequence:** Increased friction, workarounds and risk of errors.

Understaffing & pace – (risk: A−, C−, R−)

**• Support:** Minimum safe staffing, appropriate coverage and realistic workload.

**• Thwarting:** Chronic staffing gaps and insufficient coverage.

**• Consequence:** Workarounds, strain and reduced capacity to maintain a sustainable pace.

Parallel systems to compensate – (risk: C−, A−)

**• Support:** A consolidated single source of truth.

**• Thwarting:** Shadow lists, parallel documentation and compensatory information systems.

**• Consequence:** Mismatch, duplication and rework.

Poor planning/system collapse – (risk: C−, A−, R−)

**• Support:** Reliable coordination, predictable planning and sufficient capacity to accommodate variation.

**• Thwarting:** Repeated last-minute changes and planning that assumes ideal conditions.

**• Consequence:** Improvisation, instability, workarounds and blame.

Unsustainable tempo/production chase – (risk: A−, C−)

**• Support:** Quality-paced operating lists with sufficient room to maintain standards of care.

**• Thwarting:** Speed or throughput treated as a standing priority regardless of contextual constraints.

**• Consequence:** Corner-cutting and reduced capacity to maintain professional standards.

Work organisation & structure

Resources & ways of working – (C+, A+ | risk: C−, A−)

**• Support:** Appropriate skill mix, standardised working and sufficient flexibility to respond to changing demands.

**• Thwarting:** Staffing gaps, ad-hoc reallocations and continual firefighting.

**• Consequence:** Stable tempo and effective use of competence versus reactive scrambling.

Technical resources & equipment – (C+ | risk: C−)

**• Support:** Maintained and accessible equipment, local availability of essential resources and rapid resolution of faults.

**• Thwarting:** Equipment failures, shared bottlenecks and delays while waiting for replacement or alternative equipment.

**• Consequence:** Confident execution versus delays, interruptions and risk of error.

Structure & decision-making – (A+, R+ | risk: A−, R−)

**• Support:** Voice in planning, shared rationales and brief pre-list coordination or huddles.

**• Thwarting:** Opaque or late changes made without context or meaningful staff involvement.

**• Consequence:** Ownership and engagement versus disengaged compliance.

Workflow & efficiency – (C+, R+ | risk: C−)

**• Support:** Smooth handoffs, standardised coordination and a single source of truth.

**• Thwarting:** Double charting, parallel notes and workflow bottlenecks.

**• Consequence:** Fewer interruptions and smoother flow versus rework.

Organisational culture – (R+, A+ | risk: R−, A−)

**• Support:** Respect, craftsmanship, learning and a shared focus on care quality.

**• Thwarting:** Cynicism and a predominant focus on meeting production targets.

**• Consequence:** Quality-focused work versus production chase.

Information handling – (A+, C+ | risk: C−)

**• Support:** Timely information, clear ownership and a single source of truth.

**• Thwarting:** Double entry, parallel sheets and contradictory information sources.

**• Consequence:** Fewer contradictions and more predictable work versus mismatch and rework.

Staffing & scheduling – (A+ | risk: A−, C−)

**• Support:** Predictable rosters, fair scheduling rules and sufficient notice of changes.

**• Thwarting:** Frequent short-notice changes and unpredictable shifts.

**• Consequence:** Recovery and perceived control versus cumulative fatigue and reduced predictability.

Time/resources for quality – (A+, C+)

**• Support:** Realistic operating lists and sufficient buffer for checks, preparation and unexpected variation.

**• Thwarting:** Planning without margins or slack.

**• Consequence:** Craftsmanship and maintained standards versus shortcuts.

Main category – Psychologically safe teamwork

Teamwork & communication

Collegial support & tacit coordination – (R+, C+)

**• Support:** Stable collaboration, anticipation of colleagues' needs and smooth handoffs.

**• Thwarting:** Fragmented teams and inconsistent collaboration.

**• Consequence:** Fewer corrections and smoother coordination versus repeated resets.

Destructive communication & insecurity – (risk: R−, A−)

**• Support:** Civil, no-blame correction and respectful feedback.

**• Thwarting:** Sarcasm, blame and sharp communication, particularly under pressure.

**• Consequence:** Psychological safety and timely correction versus silence and delay.

Silence – imposed/normalised – (risk: A−, C−, R−)

**• Support:** Explicit speak-up norms and routine opportunities to raise concerns.

**• Thwarting:** Experiences of retaliation, futility or a perception that raising concerns is not worthwhile.

**• Consequence:** Earlier correction of risk versus uncorrected hazards and delayed escalation.

Power, hierarchy & leadership

Hierarchical roles & status differences – (risk: A−, R−)

**• Support:** Clear professional roles without loss of voice or influence.

**• Thwarting:** Deference and status gradients that inhibit speaking up.

**• Consequence:** Professional initiative versus hesitation.

Hierarchy lowers safety – (risk: A−, C−, R−)

**• Support:** Psychological safety embedded in briefings and everyday interaction.

**• Thwarting:** Public put-downs, dismissive responses and delayed correction.

**• Consequence:** Real-time correction and learning versus error carry-over.

Respectful team culture & parity – (R+, A+)

**• Support:** Equitable voice across professional roles and respectful dialogue.

**• Thwarting:** Dominance, dismissal and steep status gradients.

**• Consequence:** Shared problem-solving versus disengagement.

Teamwork & social work environment

Basic cohesion & group dynamics – (R+, C+ | risk: R−)

**• Support:** Stable pairings, tacit handoffs and clear roles.

**• Thwarting:** Frequent rotation, conflict cycles and uneven workload distribution.

**• Consequence:** Smoother operating lists versus repeated resets after disruptions.

Collegial relationships & closeness – (R+, A+ | risk: R−)

**• Support:** Approachability, easy help-seeking and proactive backup from colleagues.

**• Thwarting:** Social distance and reluctance to seek assistance.

**• Consequence:** Earlier escalation versus silent coping.

Climate & enjoyment – (R+, A+ | risk: R−)

**• Support:** Civility, humour and a consistently respectful tone.

**• Thwarting:** Sarcasm, eye-rolling and tone that deteriorates under load.

**• Consequence:** Greater learning bandwidth versus emotional drain.

Basic collegial support & reciprocity – (R+, C+ | risk: R−, C−)

**• Support:** Reciprocal helping norms, practical support and shared responsibility, particularly during stress and on-call work.

**• Thwarting:** Uneven helping, uncertainty among new staff and limited cross-professional backup.

**• Consequence:** Stress buffering, smoother flow and competence development versus friction and slower learning.

Social activities & belonging – (R+ | risk: R−)

**• Support:** Shared breaks, informal rituals and opportunities for informal debriefing.

**• Thwarting:** Split breaks and limited shared spaces or activities.

**• Consequence:** Stronger belonging and smoother coordination versus brittle handoffs.

Teamwork challenges – (risk: A−, R−, C−)

**• Support:** Active conflict moderation and fair allocation of workload.

**• Thwarting:** Status games, blame loops and unresolved interpersonal conflict.

**• Consequence:** Reduced silence and fewer workarounds versus under-reporting and disengagement.

Interprofessional communication – (R+, C+ | risk: R−)

**• Support:** Short communication loops, accessibility and respectful tone.

**• Thwarting:** Sharpness, inaccessibility and reluctance to initiate communication across professional boundaries.

**• Consequence:** Earlier corrections versus hesitation.

(Inter-)professional values & stance – (R+, A+)

**• Support:** Shared respect and human-centred care values.

**• Thwarting:** Role contempt, territorial behaviour and professional dismissal.

**• Consequence:** Smooth collaboration versus friction.

Patient focus & quality – (A+, C+, R+ | risk: A−, C−)

**• Support:** Patient-centred decision-making and sufficient time to maintain care quality.

**• Thwarting:** Production pressure that displaces patient-centred priorities.

**• Consequence:** Purposeful care versus corner-cutting.

Leadership & occupational health and safety responsibility – (A+, R+ | risk: R−)

**• Support:** Accessible leadership, visible hazard removal and timely response to recurring barriers.

**• Thwarting:** Delayed responses and low leadership visibility.

**• Consequence:** Safe voice and confidence that concerns matter versus learned silence.

Main category – Participatory planning & visible follow-through

Lack of influence & participation – (risk: A−)

**• Support:** Staff input has visible consequences for planning and operating lists.

**• Thwarting:** Consultation in which decisions are already effectively settled.

**• Consequence:** Ownership versus compliance.

Production over care – (risk: A−, C−)

**• Support:** Performance measures balanced with care quality and professional meaning.

**• Thwarting:** Numerical output taking precedence over care values.

**• Consequence:** Maintenance of craft quality versus corner-cutting.

Engaged leadership & change capacity – (A+, C+, R+)

**• Support:** Barrier removal, co-creation and rapid, visible responses to problems raised by staff.

**• Thwarting:** Tokenistic participation and slow or unclear follow-through.

**• Consequence:** Ownership and change capacity versus participation fatigue.

Quality improvement & patient safety – (C+, R+ | risk: A−)

**• Support:** Frontline-led quality-improvement initiatives, opportunities to test small changes and visible feedback loops.

**• Thwarting:** Top-down audits or improvement initiatives without meaningful feedback or influence.

**• Consequence:** Ownership and learning versus initiative fatigue.

Historical development – (A+ | risk: A−)

**• Support:** Visible practical improvements and credible evidence that previous input has led to change.

**• Thwarting:** Repeated unsuccessful initiatives and perceptions that earlier attempts produced no meaningful improvement.

**• Consequence:** Engagement versus scepticism and tokenism.

Strategic perspective – (A+, R+ | risk: A−)

**• Support:** A shared rationale for organisational aims and explicit alignment with patient-care values.

**• Thwarting:** A predominantly metrics-based framing of performance.

**• Consequence:** Commitment and shared purpose versus compliance.

Main category – Learning structures to mastery; load as boundary

Learning & professional development

Supervision/onboarding gaps – (risk: C−, A−)

**• Support:** Structured onboarding, buddying and scaffolded progression towards independent practice.

**• Thwarting:** Variable or “sink-or-swim” introduction.

**• Consequence:** Confident progression versus anxiety and prolonged dependence.

Missing space for reflection – (risk: C−, R−)

**• Support:** Brief reflective huddles, guided debriefing and psychologically safe review of adverse events or near-misses.

**• Thwarting:** Lack of time for reflection or blame-oriented review.

**• Consequence:** Integration and learning versus defensive routines.

Undervalued/underused expertise – (risk: C−, A−)

**• Support:** Role-fit assignments, recognition of expertise and continuity that allows specialist competence to be used.

**• Thwarting:** Misassignment or failure to make use of available expertise.

**• Consequence:** Engagement and professional efficacy versus withdrawal.

Stress, safety & well-being

Moral distress/guilt & rumination – (risk: A−, R−)

**• Support:** Opportunities for debriefing and support following ethically or emotionally difficult situations.

**• Thwarting:** Lack of time or forums in which experiences can be processed.

**• Consequence:** Integration versus persistent guilt and rumination.

Less patient contact/erosion of safety – (risk: R−, C−)

**• Support:** Protected time for patient contact and clinically meaningful interaction.

**• Thwarting:** Throughput pressure that reduces such contact.

**• Consequence:** Relational reward and maintained care quality versus a more transactional experience of care.

Stress impact on care quality – (risk: C−)

**• Support:** Buffer staffing, realistic pacing and sufficient slack to absorb variability.

**• Thwarting:** Stacked workload and insufficient margin for unexpected events.

**• Consequence:** Pride in workmanship and maintained quality versus slips and compromised standards.

Physical & mental exhaustion – (risk: A−, C−, R−)

**• Support:** Relief, protected breaks and recovery norms.

**• Thwarting:** Long periods of sustained workload and missed breaks.

**• Consequence:** Energy for learning and engagement versus detachment and exhaustion.

Cynicism/loss of spark – (risk: A−, R−)

**• Support:** Visible improvements, recognition and evidence that recurring problems can be resolved.

**• Thwarting:** Persistent organisational barriers.

**• Consequence:** Engagement versus cynicism and emotional armour.

Competence, development & sustainability

Recruitment & turnover – (R+, C+ | risk: R−, C−)

**• Support:** Stable teams, retention and preservation of tacit knowledge.

**• Thwarting:** High turnover and continual need to rebuild competence.

**• Consequence:** Consistency versus perpetual onboarding.

Induction & basic training – (C+, A+ | risk: C−)

**• Support:** Structured onboarding, buddying, skills ladders and supervised autonomy.

**• Thwarting:** Short or variable induction with inconsistent exposure.

**• Consequence:** Faster progression towards independent practice versus prolonged dependence.

Competence & professional growth – (C+, A+ | risk: C−)

**• Support:** Deliberate practice, recognition and protected time for skill development.

**• Thwarting:** Learning and development repeatedly displaced by workload.

**• Consequence:** Self-efficacy and mastery versus stagnation.

Pride & work ethic – (A+, C+, R+ | risk: A−)

**• Support:** Professional standards and craftsmanship experienced as personally meaningful.

**• Thwarting:** Chronic constraints that require repeated compromise of professional standards.

**• Consequence:** Discretionary effort and pride versus minimalism.

Working hours & flexibility – (A+ | risk: A−, C−)

**• Support:** Fair flexibility and opportunities for staff influence over working patterns.

**• Thwarting:** Rigid scheduling and accumulating overtime.

**• Consequence:** Recovery and perceived autonomy versus cumulative fatigue.

Continuing education & knowledge sharing – (C+, R+ | risk: C−)

**• Support:** Protected learning time, peer teaching and regular opportunities for knowledge sharing.

**• Thwarting:** Irregular educational opportunities and load-related cancellation.

**• Consequence:** Capability growth and shared competence versus skill drift.

Sustainability signals: motivation and retention

Stress & workload – (risk: A−, C−, R−)

**• Support:** Protected pauses, relief capacity and opportunities for recovery.

**• Thwarting:** Chronic overload with insufficient recovery.

**• Consequence:** Fatigue, narrowed attention and moral strain.

Joy at work & motivation – (A+, R+, C+)

**• Support:** Experiences of flow, meaningful patient connection and opportunities to exercise professional competence.

**• Thwarting:** Persistent time pressure that constrains these experiences.

**• Consequence:** Engagement versus going through the motions.

Challenges & dissatisfaction – (risk: A−, R−, C−)

**• Support:** Timely solutions to recurring practical problems and visible feedback following concerns.

**• Thwarting:** Persistent barriers without an effective feedback loop.

**• Consequence:** Engagement versus cynicism and detachment.

Stay if conditions improve vs consider exit – (A+ | risk: A−, C−)

**• Support:** Credible plans to improve fundamental working conditions.

**• Thwarting:** Expectations that recurring problems will remain unchanged.

**• Consequence:** Conditional commitment versus consideration of leaving.

Love of the profession persists – (R+, C+)

**• Support:** Strong professional identity, purpose and pride in clinical craft.

**• Thwarting:** Conflict between professional identity and organisational conditions.

**• Consequence:** Professional meaning as a buffer versus erosion of motivation.

Meaning & human-centred care – (A+, R+, C+)

**• Support:** Purpose-anchored routines and explicit connection between organisational work and patient care.

**• Thwarting:** Performance framing dominated by metrics alone.

**• Consequence:** Intrinsic motivation versus motivational drift.
